# Epidemiology of Invasive Fungal Diseases in Patients with Hematologic Malignancies and Hematopoietic Cell Transplantation Recipients Managed with an Antifungal Diagnostic Driven Approach

**DOI:** 10.3390/jof7080588

**Published:** 2021-07-23

**Authors:** Maria Daniela Bergamasco, Carlos Alberto P. Pereira, Celso Arrais-Rodrigues, Diogo B. Ferreira, Otavio Baiocchi, Fabio Kerbauy, Marcio Nucci, Arnaldo Lopes Colombo

**Affiliations:** 1 Division of Infectious Diseases, Hospital São Paulo-University Hospital, Universidade Federal de São Paulo, São Paulo 04024-002, Brazil; mdanielabergamasco@gmail.com (M.D.B.); capp1708@gmail.com (C.A.P.P.); diogobferreira@gmail.com (D.B.F.); 2 Division of Hematology, Hospital São Paulo-University Hospital, Universidade Federal de São Paulo, São Paulo 04024-002, Brazil; celsoarrais@gmail.com (C.A.-R.); obaiocchi@gmail.com (O.B.); fkerbauy@gmail.com (F.K.); 3 Department of Internal Medicine, Hospital Universitário Clementino Frafa Filho, Universidade Federal do Rio de Janeiro, Rio de Janeiro 21941-913, Brazil; mnucci@hucff.ufrj.br

**Keywords:** fungal infection, invasive fungal disease, hematologic malignancy, hematopoietic cell transplantation, epidemiology, aspergillosis, fusariosis, candidiasis, cryptococcosis

## Abstract

Patients with hematologic malignancies and hematopoietic cell transplant recipients (HCT) are at high risk for invasive fungal disease (IFD). The practice of antifungal prophylaxis with mold-active azoles has been challenged recently because of drug–drug interactions with novel targeted therapies. This is a retrospective, single-center cohort study of consecutive cases of proven or probable IFD, diagnosed between 2009 and 2019, in adult hematologic patients and HCT recipients managed with fluconazole prophylaxis and an antifungal diagnostic-driven approach for mold infection. During the study period, 94 cases of IFD occurred among 664 hematologic patients and 316 HCT recipients. The frequency among patients with allogeneic HCT, autologous HCT, acute leukemia and other hematologic malignancies was 8.9%, 1.6%, 17.3%, and 6.4%, respectively. Aspergillosis was the leading IFD (53.2%), followed by fusariosis (18.1%), candidiasis (10.6%), and cryptococcosis (8.5%). The overall 6-week mortality rate was 37.2%, and varied according to the host and the etiology of IFD, from 28% in aspergillosis to 52.9% in fusariosis. Although IFD occurred frequently in our cohort of patients managed with an antifungal diagnostic driven approach, mortality rates were comparable to other studies. In the face of challenges posed by the use of anti-mold prophylaxis, this strategy remains a reasonable alternative.

## 1. Introduction

Patients with hematologic malignancies are at high risk of developing invasive fungal disease (IFD). The highest incidences have been reported in allogeneic hematopoietic cell transplant (HCT) recipients and in patients with acute myeloid leukemia (AML) receiving induction remission chemotherapy [1,2,3,4,5,6]. In addition, recent studies have shown a high incidence of IFD in patients with acute lymphoid leukemia (ALL) [7,8], and the emergence of a new group at risk: patients with chronic lymphoproliferative diseases receiving ibrutinib [9,10,11].

The incidence and etiologic agents of IFD differ across regions in the globe. These geographic variations may reflect differences in the population of patients at risk, practices of antifungal prophylaxis and environmental exposure [4,6,12,13]. Most recent studies addressing the landscape of IFD in hematologic patients included a large number of patients receiving antifungal prophylaxis with a mold-active agent, especially posaconazole and voriconazole [14]. In recent years, several new drugs have been incorporated in the treatment of AML [15] and graft versus host disease (GVHD) [16,17]. While the introduction of these targeted therapies represents a great advance in the field, with significant improvements in the outcome, the use of mold-active azoles as prophylaxis may be problematic because of drug–drug interactions. Both posaconazole and voriconazole are strong cytochrome P450 3A4 inhibitors [18,19], which is the main metabolic pathway of these targeted therapies [20]. In addition, prolongation of the QT interval by both azoles may increase the risk of side effects. Therefore, a move back to fluconazole prophylaxis is likely to occur if one of these targeted therapies is used. In the present study, we describe the epidemiology of IFD in patients with hematologic diseases treated at a single center in Brazil, managed with fluconazole prophylaxis and a diagnostic-driven antifungal approach for mold infections.

## 2. Materials and Methods

### 2.1. Study Design, Population and Data Collection

This is a retrospective cohort study conducted at Hospital São Paulo, a tertiary-care public university hospital of the Universidade Federal de São Paulo, Brazil. We included all consecutive cases of IFD occurring in adult patients (≥16 years-old) with hematologic malignancies or undergoing HCT between 2009 and 2019 (11-year period). The study was approved by the local Ethics Committee (number 2.726.506/2018). Only cases of proven or probable IFD according to the European Organization for Research and Treatment of Cancer/Mycosis Study Group—EORTC/MSG [21] were included. Cases of *Pneumocystis jirovecii* pneumonia were excluded. If a patient developed more than one episode of IFD, only the first episode was considered in the analysis.

The patient’s medical charts were reviewed for demographic characteristics, underlying disease and recent treatment, transplant details including donor type, conditioning regimen, stem cell source and graft versus host disease, clinical and radiological aspects, mycological results, including galactomannan antigen tests, antifungal therapy and 6-week survival. Data were collected using a case report form with a dictionary of terms. All data were recorded in a SPSS file (SPSS Statistics v.24.0, IBM, São Paulo, Brazil).

### 2.2. Screening, Diagnostic Procedures for IFD and Antifungal Practices

Patients were cared for in rooms without an HEPA filter, except for HCT recipients, who were cared for in rooms with an HEPA filter and positive pressure, which has occurred since 2012. Patients with acute leukemia and HCT recipients received primary antifungal prophylaxis with fluconazole during neutropenia (until D+75 for allogeneic HCT). Screening for IFD included blood cultures in the case of fever, and monitoring with a serum galactomannan antigen test, performed three times a week during neutropenia and once a week in allogeneic HCT recipients with grade 3–4 GVHD. Chest and sinuses computed tomography (CT) was performed in the case of persistent or recurrent fever during neutropenia or if patients presented with respiratory symptoms. In addition, if the patient presented with clinical or radiological manifestations suspicious of IFD, serum galactomannan was obtained for 2–3 consecutive days. This galactomannan-based diagnostic-driven strategy was applied during the whole study period. Other tests performed by clinical indication included bronchoscopy with bronchoalveolar lavage (BAL), sputum culture and skin biopsy.

Galactomannan antigen testing was performed using the Platelia™ Aspergillus Ag (Bio-Rad^®^,Marnes-la-Coquette, France and Lagoa Santa, Brazil) kit, and was considered positive with 2 consecutive values ≥0.5 or a single value ≥0.7 in the serum, and ≥1.0 in the BAL. All fungal isolates growing in culture were identified at species level based on their micromorphological characteristics and sequencing of an informative DNA target, as previously described [22,23].

Neutropenia was defined as an absolute neutrophil count (ANC) <500/mm^3^ and recovery from neutropenia (or engraftment in HCT) as ANC >500/mm^3^ in at least 3 consecutive blood counts. Fever was defined as an axillary temperature >38 °C.

### 2.3. Descriptive and Statistical Analysis

The rates of IFD overall and for different underlying conditions were calculated using the number of patients treated in the study period as the denominator. The date of the diagnosis of IFD was defined as the date of the first clinical documentation of infection. We compared patients’ characteristics and the rates of IDF among three groups of underlying conditions and/or treatments: HCT, acute leukemia and other underlying diseases. Categorical variables were compared by using the Pearson chi-square test or Fisher’s exact test as appropriate, and continuous variables were compared using Mann–Whitney or Kruskal–Wallis tests, as appropriate. Kaplan–Meier curves were constructed to evaluate the 6-week survival, and were compared using the log-rank test. All tests were 2-tailed and a *p* value < 0.05 was considered statistically significant. All statistical analysis was performed using the SPSS Statistics v.24.0 (IBM, São Paulo, Brazil, license number 10101151149).

## 3. Results

### 3.1. Frequency and General Characteristics of Patients with IFD

During the study period, 94 cases of IFD (50 proven and 44 probable) were diagnosed in 94 patients. The characteristics of these 94 patients are shown in Table 1. There were 75 cases of IFD among 664 patients with hematologic malignancies (11.3%), and 19 cases among 316 HCT (6.0%). The frequency of IDF among patients with allogeneic HCT, autologous HCT, acute leukemia and other hematologic malignancies was 8.9% (17/191), 1.6% (2/125), 17.3% (51/294), and 6.4% (24/372), respectively.

The most frequent IFD was aspergillosis, with 50 of the 94 cases (53.2%), followed by fusariosis (17 cases, 18.1%), candidiasis (10 cases, 10.6%), and cryptococcosis (eight cases, 8.5%). The other nine cases were represented by hyalohyphomycosis (*n* = 4), mucormycosis and infection by *Penicillium* sp. (two cases each), and trichosporonosis (*n* = 1). The sites of infection among the 50 cases of aspergillosis were lungs (*n* = 38), lungs and sinuses (*n* = 8), sinuses (*n* = 3) and central nervous system (CNS, *n* = 1). Among 10 cases of aspergillosis with species identification, *A. fumigatus* was the most frequent (*n* = 6), followed by *A. flavus* (*n* = 4).

Fusariosis involved the skin in 10 of the 17 cases, lungs in 13, and bloodstream in 8 cases. The most frequent clinical presentation was lungs, skin and bloodstream (*n* = 3). All 17 cases were caused by *Fusarium solani* species complex.

Nine of the ten cases of candidiasis were bloodstream infections and there was one case of peritoneal candidiasis. The species were *Candida albicans* (*n* = 4), *Candida parapsilosis* (*n* = 3), *Candida krusei* (*n* = 2) and *Candida glabrata* (*n* = 1).

All cases of cryptococcosis were caused by *Cryptococcus neoformans.* Six of the eight cases of were fungemia, one involved the lungs and one lungs and CNS.

Table 2 shows the distribution of IFD in the four groups. Aspergillosis was the most frequent IFD in all groups. Fusariosis was the second most frequent IFD in allogeneic HCT and acute leukemia, whereas in the group of other underlying diseases, cryptococcosis was the second most frequent IFD, with seven cases. These seven cases occurred in patients with lymphoproliferative diseases: five in patients with non-Hodgkin’s lymphoma and one each in patients with Hodgkin’s lymphoma and chronic lymphoid leukemia. Indeed, cryptococcosis was significantly more frequent in patients with lymphoproliferative diseases compared with the other three groups (*p* < 0.001).

The majority of patients (76, 80.9%) developed IFD while on fluconazole prophylaxis: 76.5% of allogeneic HCT recipients, 92.2% of patients with acute leukemia, 58.3% of patients with other hematologic conditions and the two autologous HCT recipients. Seven of the ten cases of candidemia and one of the eight cases of cryptococcosis occurred in patients receiving fluconazole prophylaxis. *Candida* species occurring in breakthrough infection under fluconazole prophylaxis were *C. albicans*, *C. parapsilosis* and *C. krusei* (two cases each), and *C. glabrata* (one case).

In 40 of the 50 cases of aspergillosis the disease was classified as probable. The basis for the diagnosis in these 40 cases was a combination of imaging and positive galactomannan in the serum (*n* = 34), BAL (*n* = 3) or both (*n* = 3). Among the 37 cases with positive serum galactomannan, the median peak value was 0.944 (range, 0.505–6.302) and the median number of positive tests was two (range 1–15).

### 3.2. Characteristics of Patients with IFD in HCT Recipients

**Figure 1:**
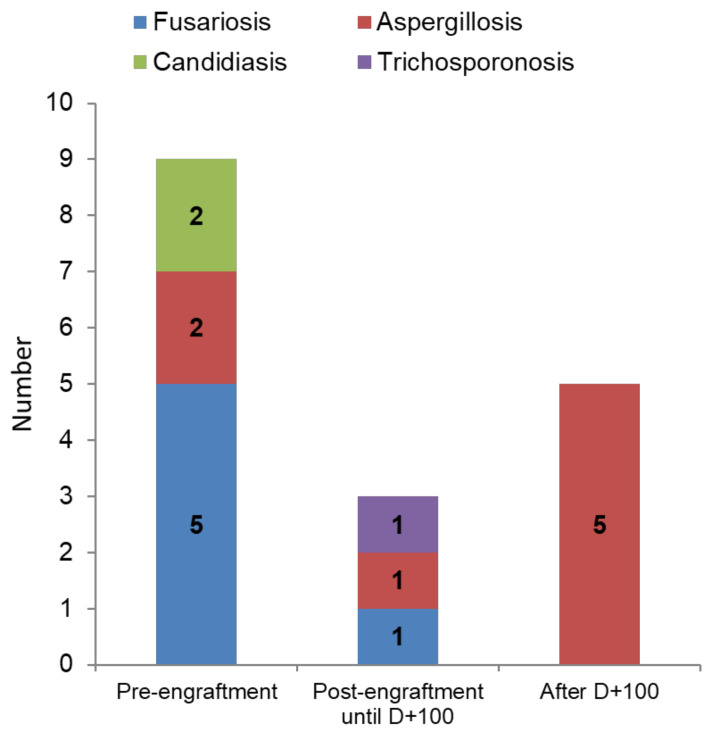
Distribution of invasive fungal disease in allogeneic hematopoietic cell transplant recipients according to the period post-transplant.

The frequency of IFD among different types of allogeneic HCT was as follows: 7.0% (8/115) for matched-related donor, 17.9% (5/28) for matched-unrelated donor, and 8.3% (4/48) for haplo-identical HCT. In allogeneic HCT recipients, IFD occurred at a median of 40 days after transplant (range, 7–2350 days). Nine of the seventeen cases of IFD (52.9%) occurred in the pre-engraftment period, at a median of 15 days post-transplant (range, 7–40). Three cases (17.6%) occurred between engraftment and day +100 (median day +78), and the remaining five cases (29.4%) occurred after day +100 (median day +239). Figure 1 shows the distribution of the different IFD in these three periods.

The underlying diseases in the 17 patients were as follows: AML (*n* = 5), ALL and aplastic anemia (three each), chronic lymphoid leukemia, and myelodysplasia (two each), and chronic myeloid leukemia and multiple myeloma (one each). The transplants included HLA-matched sibling donors in eight patients, HLA-matched unrelated donors in five, and haplo-identical in four patients.

Neutropenia at the time of diagnosis of IFD was present in all cases occurring in the pre-engraftment period, two of three (66.7%) until day +100, and one of five (20%) after day +100. By contrast, GVHD occurred in 22.2% in the pre-engraftment period, 33.3% until day +100, and 100% after day +100 (*p* = 0.02). The 6-week mortality was 44.4% in cases occurring in the pre-engraftment period, 33.3% until day +100, and 40% after day +100 (*p* = 0.94). The 6-week mortality rates per IFD were: 25% in aspergillosis, 50% in fusariosis and 50% in candidiasis. The single patient with invasive trichosporonosis died.

Two cases of IFD were diagnosed in autologous HCT recipients: a patient with multiple myeloma who developed candidiasis on day +1 post-transplant and a patient with Hodgkin’s lymphoma with aspergillosis diagnosed on day +17. Both patients survived.

### 3.3. Characteristics of Patients with IFD in Acute Leukemia

Among the 51 cases of IFD in patients with acute leukemia, 39 occurred in 187 patients with AML (20.9%), 9 cases among 69 patients with ALL (13.0%), 2 cases in 35 patients with acute promyelocytic leukemia (APL, 5.7%), and 1 case in a patient with dendritic cell leukemia. The median age of the patients was 58 years (range 18–73) and 28 were female. The median duration of hospitalization before IFD was 27 days (range 0–147), and did not differ among patient with AML and ALL (median of 27 days in both groups).

The majority of patients with AML developed IFD during the first induction chemotherapy, with a cytarabine/anthracycline-based regimen (51.3%). Three cases occurred during consolidation therapy, and one during maintenance. The other 15 cases occurred in the setting of relapsed or refractory disease. Aspergillosis was the most frequent IFD (*n* = 22), followed by fusariosis (*n* = 5), candidiasis (*n* = 4), hyalohyphomycosis (*n* = 3), penicillinosis and mucormycosis (two each) and cryptococcosis (*n* = 1).

All patients with ALL were in first line therapy (protocol GMALL in seven patients and GRAALL in two). Six cases occurred during induction and three during consolidation. The IFDs were: aspergillosis (*n* = 4), candidiasis and fusariosis (two each), and hyalohyphomycosis (*n* = 1).

Neutropenia was present at the diagnosis of IDF in all but three patients with acute leukemia. These three cases of IFD diagnosed in non-neutropenic patients were: candidiasis in a patient with ALL receiving consolidation therapy, fungal sinusitis (hyalohyphomycosis) in a patient with ALL receiving maintenance therapy, and aspergillosis in a patient with APL after induction with retinoid acid + anthracycline. The 6-week mortality rate was 41% in patients in AML and 22% in ALL. None of the two patients with APL died. The patient with dendritic cell leukemia died. The 6-week death rate by the etiology of IFD was as follows: 17.9% in aspergillosis, 50% in fusariosis, 33.3% in candidiasis, 50% in hyalohyphomycosis, 50% in penicillinosis and 100% in mucormycosis.

### 3.4. Characteristics of Patients with IFD in Other Hematologic Diseases

The most frequent IFD in this group was aspergillosis (13 of the 24 cases, 54.2%), followed by cryptococcosis (seven cases, 29.2%), and candidiasis and fusariosis (two cases each). Most episodes occurred in patients with non-Hodgkin’s lymphoma (*n* = 10), followed by aplastic anemia (*n* = 4), Hodgkin’s lymphoma and chronic lymphoid leukemia (three each), multiple myeloma (*n* = 2), and large granular lymphocyte leukemia and myelofibrosis (one each).

Neutropenia was present in 14 of the 24 cases (58.3%), and 14 patients were receiving corticosteroids. Five patients developed IFD outside the context of neutropenia or corticosteroid use: three cases of cryptococcosis, and one each of aspergillosis and candidemia. Three of these five patients had received more than two lines of chemotherapy for the treatment of their underlying malignancy. The 6-week death rate by etiology of IFD was 28.6% in cryptococcosis, 38.5% in aspergillosis, and 50% in fusariosis and candidiasis.

### 3.5. Antifungal Treatment

Only three patients did not receive treatment: two with aspergillosis and one with cryptococcosis. As shown in Table 3, voriconazole was the most frequent agent used in the treatment of aspergillosis, either as monotherapy (19 patients) or in combination with micafungin (*n* = 7), deoxycholate amphotericin B (*n* = 2), or liposomal amphotericin B (*n* = 1). Seven of the seventeen patients with fusariosis received treatment with a combination of deoxycholate amphotericin B and voriconazole. Treatment for candidiasis was as follows: deoxycholate amphotericin B (*n* = 5), fluconazole (*n* = 3), and micafungin (*n* = 2). The median duration of antifungal therapy was 34 days (range 11–126).

### 3.6. Overall 6-Week Outcomes

**Figure 2:**
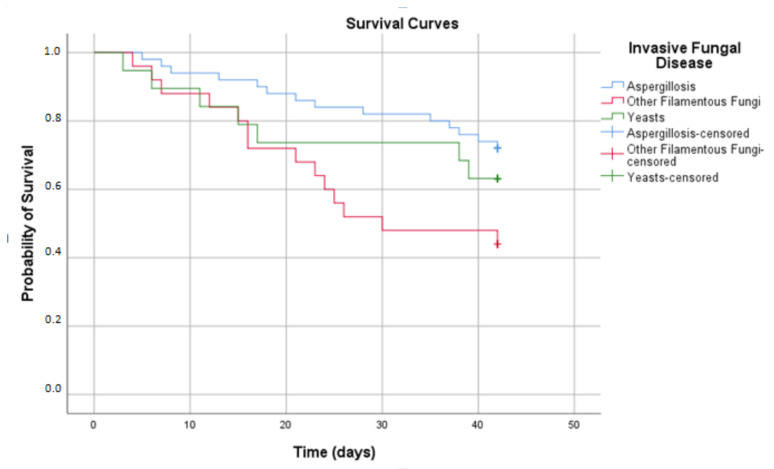
Six-week survival curves for all patients (all underlying conditions) according to the type of invasive fungal disease (log rank, *p* = 0.04).

The overall 6-week mortality rate in the cohort of 91 patients with IFD was 37.2%. The mortality rates varied according to the underlying condition, as described above. Considering the different etiologies of IFD, the 6-week mortality was lower in patients with aspergillosis (28.0%), candidiasis (30%) and cryptococcosis (37.5%) compared with infection caused by other filamentous fungi (52.9% in fusariosis and 50.0% in other hyalohyphomycoses). Both patients who developed mucormycosis and the single patient with trichosporonosis died. Figure 2 shows the survival curves by the etiology of IFD.

## 4. Discussion

In this retrospective, single-center study, we evaluated the epidemiology of IFD in patients with hematologic malignancies and HCT recipients receiving fluconazole prophylaxis and anti-mold antifungal therapy guided by diagnostic screening with galactomannan and CT scans. The overall frequency of IFD was 11.7%, being highest in AML (20.9%) and ALL (13.0), followed by allogeneic HCT (8.9%). Aspergillosis was the most frequent IFD.

As already reported in other epidemiologic studies of IFD in patients with hematologic malignancies [5,6,8,24,25], in our study, AML was the underlying condition associated with the highest frequency of IFD. The 20.9% rate observed is in the top range of 10‒17% described in other studies [5,6,26,27], and similar to the incidence reported in a recent study from Brazil, in which patients also received fluconazole prophylaxis and were monitored with serial serum galactomannan and chest CT scan [8]. The high burden of IFD in Brazilian centers when compared to other countries may reflect suboptimal environmental protection, as most patients with AML receive induction remission in rooms without an HEPA filter and positive pressure. Another possible explanation is the option for fluconazole rather than posaconazole as prophylaxis.

An interesting finding of our study was the lower incidence of IFD in patients with APL (5.7%). A multicenter study conducted in Italy compared the incidence of IFD in patients with AML receiving standard induction chemotherapy with patients with APL receiving retinoid acid plus chemotherapy or retinoid acid plus arsenic trioxide. The incidence of FD was 9% in AML and 4% in APL [28]. The use of differentiating agents (retinoic acid, arsenic trioxide) in the induction remission of APL results in a lower incidence of gastrointestinal mucositis and shorter duration of neutropenia compared with a standard “7 + 3” induction regimen of AML [29,30].

In the present study, we observed a high incidence of IFD in ALL (13.0%), similar to that observed in the study by Souza et al. [8]. The incidence of IFD in ALL may vary widely, and is higher in patients receiving more intensive chemotherapeutic regimens, given to treat high-risk groups and relapsed ALL [31].

Among HCT recipients, the highest frequency of IFD was observed in matched-unrelated allogeneic HCT, as previously described and expected for HCT from alternative donors [3,4]. Regarding haplo-identical HCT, using reduced-intensity conditioning regimen and post-transplant cyclophosphamide, as previously described [32], the frequency of IFD in our study was similar to that of matched-related allogeneic HCT (8.3% and 7.0%, respectively). This is in agreement with studies showing a much higher frequency of IFD among haplo-identical HCT T-cell depleted versus T-cell replete transplant with post-transplant cyclophosphamide [33].

In agreement with other studies evaluating the epidemiology of IFD in hematology [3,4,5,6,8,24,33], aspergillosis was the most frequent IFD in our study. In addition, similar to other studies conducted in Brazil [6,8], fusariosis was the second most frequent IFD. Fusariosis present peculiarities in terms of its geographical distribution in hematological patients, being less frequent in European countries when compared to Brazilian centers [34,35]. Our data also confirm that infection caused by agents of mucormycosis is rare in Brazilian hematologic patients [6], in sharp contrast with its higher frequency in other regions of the globe [4]. In addition, cryptococcosis was the fourth most common IFD (8.5%) and, as expected, occurred almost exclusively in patients with lymphoproliferative diseases, in general after multiple lines of treatment [36].

Due to resource limitations in our hospital, a substantial proportion of patients with IFD received deoxycholate amphotericin B as treatment, either alone (29 patients) or in combination with other agents (13 patients, Table 3). Despite these shortcomings, the overall 6-week mortality rate was similar to that reported in other studies. For example, the 52.9% mortality rate in fusariosis is comparable with that reported in a large multicenter retrospective study [37]. Similarly, the mortality rate in aspergillosis (28%) is similar to that reported in other studies [26,38], and provides additional support to the strategy of anti-*Candida* prophylaxis with fluconazole and early antifungal therapy with a mold-active agent driven by intensive surveillance and diagnostic workup. As reported in randomized studies comparing empiric versus preemptive (diagnostic-driven) antifungal therapy, the latter strategy is associated with a higher rate of microbiologic documentation of IFD but similar mortality rates [39,40].

Antifungal prophylaxis with mold-active azoles in high-risk hematologic patients is the standard of care in patients with AML and in allogeneic HCT recipients with severe GVHD [41,42]. In recent years, new and very active drugs have been incorporated in the treatment of AML (e.g., midostaurin, gilteritinib, venetoclax) and GVHD (e.g., ibrutinib, ruxolitinib) [15,16,17]. These compounds may have important drug interactions with mold-active azoles, especially posaconazole and voriconazole, including the inhibitory action of azoles on CYP3A4, and QT prolongation [43]. Fluconazole is a moderate CYP3A4 inhibitor, and its use with targeted therapies implies less dose adjustments. As shown in our study, anti-mold antifungal therapy guided by diagnostic screening with galactomannan and CT scan remains a reasonable alternative. However, given the high incidence of IFD observed in our study, the higher cost of primary anti-mold prophylaxis should be balanced with the costs of treatment and the negative impact of IFD on patients’ outcomes. An attractive alternative for anti-mold prophylais is isavuconazole, which is a moderate CYP3A4 inhibitor, does not prolong the QT interval and may exhibit fewer drug–drug interactions. However, its role as an antifungal prophylaxis is yet to be established [44,45]. Therefore, the use of anti-mold prophylaxis in these scenarios is challenging.

## 5. Conclusions

In conclusion, the frequency of IFDs in our cohort of hematologic patients managed with fluconazole prophylaxis and an antifungal diagnostic driven approach was high, but with mortality rates comparable with other studies. In the face of challenges posed by the use of anti-mold prophylaxis, this strategy still remains a reasonable alternative.

## Figures and Tables

**Table 1:** Characteristics of 94 patients with invasive fungal disease.

Characteristic	No.
Gender male:female	50:44
Age, median (range)	50.5 (16–78)
Underlying disease	
Acute myeloid leukemia	46 (48.9)
Acute lymphoid leukemia	12 (12.8)
Non-Hodgkin’s lymphoma	10 (10.6)
Aplastic anemia	7 (7.4)
Chronic lymphoid leukemia	5 (5.3)
Hodgkin’s lymphoma	4 (4.3)
Multiple myeloma	4 (4.3)
Other *	6 (6.4)
Hematopoietic cell transplantation	19 (20.2)
Autologous	2/19 (10.5)
Allogeneic	17/19 (89.5)
Matched HLA, related donor	8/17 (47.0)
HLA-matched, unrelated donor	5/17 (29.4)
Haploidentical	4/17 (23.6)
Receipt of corticosteroids within 30 days of IFD	38 (40.4)
Receipt of other immunosuppressive agents	16 (17.0)

Number in parenthesis represent percentage unless specified; * other underlying disease: myelodysplasia (*n* = 2), chronic myeloid leukemia, dendritic cell leukemia, myelofibrosis, large granular lymphocyte leukemia (1 patient each); HLA = human leukocyte antigen; IFD = invasive fungal disease.

**Table 2:** Frequency and etiology of invasive fungal disease in 316 hematopoietic cell transplant recipients and in 664 patients with hematologic malignancies.

Invasive Fungal Disease	Allogeneic HCT *n* = 191 (%)	Autologous HCT *n* = 125 (%)	Acute Leukemia *n* = 294 (%)	Other Underlying Diseases *n* = 370 (%)	Total *n* = 980 (%)
Aspergillosis	8 (4.2)	1 (0.8)	28 (9.5)	13 (3.5)	50 (5.1)
Fusariosis	6 (3.1)	0	9 (3.1)	2 (0.5)	17 (1.7)
Candidiasis	2 (1.0)	1 (0.8)	5 (1.7)	2 (0.5)	10 (1.0)
Cryptococcosis	0	0	1 (0.3)	7 (1.9)	8 (0.8)
Hyalohyphomycosis	0	0	4 (1.4)	0	4 (0.4)
Mucormycosis	0	0	2 (0.7)	0	2 (0.2)
Penicillinosis	0	0	2 (0.7)	0	2 (0.2)
Trichosporonosis	1 (0.5)		0	0	1 (0.1)
Total	17 (8.9)	2 (1.6)	51 (17.3)	24 (6.5)	94 (9.6)

HCT = hematopoietic cell transplantation.

**Table 3:** Antifungal therapy in 91 episodes of invasive fungal disease *.

Invasive Fungal Disease	Drug	No. (%)
Aspergillosis (*n* = 48)	Voriconazole	19 (39.6)
	Deoxycholate amphotericin B	13 (27.1)
	Voriconazole + micafungin	7 (14.6)
	Other **	9 (18.7)
Fusariosis (*n* = 17)	Voriconazole + deoxycholate amphotericin B	7 (41.2)
	Voriconazole	4 (23.6)
	Deoxycholate amphotericin B	3 (17.6)
	Other ***	3 (17.6)
Candidiasis (*n* = 10)	Deoxycholate amphotericin B	5 (50.0)
	Fluconazole	3 (30.0)
	Micafungin	2 (20.0)
Cryptococcosis (*n* = 7)	Deoxycholate amphotericin B	4 (57.1)
	Other ****	3 (42.9)
Hyalohyphomycosis (*n* = 4)	Deoxycholate amphotericin B	3 (75.0)
	Voriconazole	1 (25.0)
Mucormycosis (*n* = 2)	Deoxycholate amphotericin B	1
	Amphotericin B lipid complex	1
Penicillinosis (*n* = 1)	Amphotericin B lipid complex + voriconazole	1
	Voriconazole	1
Trichosporonosis (*n* = 1)	Liposomal amphotericin B + voriconazole	1

* Three patients did not receive treatment: 2 with aspergillosis and 1 with cryptococcosis; ** other treatment for aspergillosis: deoxycholate amphotericin B + voriconazole, deoxycholate amphotericin B + micafungin, and itraconazole (2 patients each), liposomal amphotericin B + voriconazole, deoxycholate amphotericin B + itraconazole, and liposomal amphotericin B (1 patient each); *** other treatment for fusariosis: liposomal amphotericin B, liposomal amphotericin B + voriconazole, and amphotericin B lipid complex + voriconazole (1 patient each); **** other treatment for cryptococcosis: itraconazole, deoxycholate amphotericin B + fluconazole, liposomal amphotericin B + fluconazole (1 patient each).
